# Insights into the heterogeneity of oculopharyngeal muscular dystrophy

**DOI:** 10.1007/s10048-025-00849-0

**Published:** 2025-09-24

**Authors:** Kyriaki Kekou, Constantinos Papadopoulos, Maria Svingou, Margarita Chrysanthou-Piterou, Evangelia Nitsa, Danai Veltra, Nikos Marinakis, Faidon-Nikolaos Tilemis, Parissis Dimitrios, Marianthi Arnaoutoglou, Maria Moschou, Sophia Xirou, Christos Bakirtzis, Georgios Tsivgoulis, Giorgos-Konstantinos Papadimas, Christalena Sofocleous

**Affiliations:** 1 Laboratory of Medical Genetics, Medical School, National and Kapodistrian University of Athens, “Aghia Sophia” Children‘s Hospital, Athens, Greece; 2 1 st Department of Neurology, Medical School, Eginition Hospital, National and Kapodistrian University of Athens, Athens, Greece; 3 Research University institute for the study and prevention of genetic and malignant disease of childhood, Athens, Greece; 4 1 st Department of Psychiatry, Medical School, Eginition Hospital, National and Kapodistrian University of Athens, Athens, Greece; 5 Department of Hygiene, Epidemiology and Medical Statistics, National and Kapodistrian University of Athens, Athens, Greece; 6 Laboratory of Genetics, Faculty of Medicine, Democritus University of Thrace, Alexandroupolis, Greece; 7 2nd Department of Neurology, Aristotle University of Thessaloniki, AHEPA Hospital, Thessaloniki, Greece; 8 1 st Department of Neurology, School of Medicine, AHEPA University Hospital, Aristotle University of Thessaloniki, Thessaloniki, Greece; 9 2nd Department of Neurology, Attikon University Hospital, National and Kapodistrian University of Athens, Athens, Greece

**Keywords:** OPMD, Triplet expansion, Anticipation, Mitochondrial dysfunction

## Abstract

Oculopharyngeal muscular dystrophy (OPMD) is a rare, adult-onset, autosomal dominant myopathy characterized by variability in the age of onset and disease progression. However, its pathogenesis and phenotypic variability remain poorly understood. The disorder is caused by an expansion of a short polyalanine tract in the poly(A) binding protein nuclear 1 *(PABPN1)* gene. This study presents data from 23 patients across 19 Greek families with pathogenic PABPN1 expansions, including demographic and laboratory data, as well as molecular and electron microscopy findings. Eight distinct trinucleotide expansion genotypes were identified. Electron microscopy consistently demonstrated mitochondrial abnormalities, including swelling, disrupted cristae and atypical lipid inclusions. Clinical heterogeneity was observed at both inter- and intrafamilial levels, and milder phenotypes were generally linked to smaller alleles. Notably, maternally inherited expansions were associated with an earlier disease onset and more severe progression in affected offspring. Given the genetic variability observed in the cohort, the presence of a founder effect could not be supported. A significant degree of underdiagnosis or diagnostic delay was noted, largely attributable to the rarity and clinical heterogeneity of the disease. The observed intrafamilial heterogeneity - particularly in maternally inherited expansions - supports previous reports suggesting that mitochondrial dysfunction may contribute to transgenerational disease progression in the context of a dominant, causative nuclear variant.

## Introduction

Oculopharyngeal muscular dystrophy (OPMD) (OMIM #164300) is an autosomal dominant myopathy typically manifesting after the fourth decade of life. It is a rare genetic disorder with a higher prevalence among French-Canadians (1:1000) [1], Jews from Bukhara (Uzbekistan) (1:700) [2], and Hispanic New Mexicans [3, 4]. The disorder is primarily characterized by ptosis and dysphagia, with or without involvement of the proximal muscles of the upper extremities, and the pelvic girdle [5]. Additional features broadening the clinical presentation include aspiration pneumonia and behavioral problems [6, 7].

The disease is mainly caused by a triplet repeat expansion in the first exon of the polyadenosine (poly[A]) binding protein nuclear 1 *(PABPN1)* gene [8]. The alanine-encoding (GCN) repeat normally contains 10 repeats and disease causing expansions lead to up to 18 alanines. The (GCN)₁₁ allele—a rather versatile variant—has been reported to: (a) be associated with either milder phenotypes or low penetrance in heterozygosity; (b) act as an aggregating modifier when in compound heterozygosity with an expanded (GCN)₁₂–₁₇ allele; and (c) cause genetically confirmed recessive OPMD in homozygosity [9–11].

The ubiquitous PABPN1 protein has a multifactorial role, being important for the regulation of poly(A) tail length and the suppression of alternative polyadenylation on mRNA transcripts [9]. However, the underlying mechanism by which specific muscle groups, such as the eyelid and pharyngeal muscles, are affected remains unclear. The primary histopathological hallmark of the disease involves the accumulation of tubulofilamentous insoluble aggregates within myonuclei of patient biopsy samples [10]. This aggregation of misfolded protein is influenced by patients’ age and genotype and has been proposed to play a detrimental role in disease progression [11]In contrast, Shademan et al. (2024), using OPMD animal and cell models as well as RNA from affected individuals, demonstrated that low levels of normal PABPN1 induce alternative splicing and production of shorter mRNA transcripts, consequently altering the muscle transcriptome [12]. Interestingly, in normal skeletal muscles, PABPN1 levels gradually decline with age, resembling protein depletion observed in OPMD affected muscles [13, 14]. Activation of the ubiquitin-proteasome system was also shown to lead to protein degradation and muscle atrophy developing independently of aggregation in both a Drosophila model and OPMD patients [15].

Mitochondrial abnormalities have been proposed as possible contributors to OPMD pathology, either as a primary feature or a downstream consequence [16, 17]. Evidence from a recent mouse model expressing a (GCN)_13_ allele suggested reduced mitochondrial metabolism [18] while abnormal accumulation of the expanded PABPN1 protein has been observed in the mitochondria of transgenic mice indicating possible potential mitochondria involvement in OPMD [17].

This study presents genetic and clinical data from OPMD patients of Greek origin, with a specific focus on a case spanning three generations of matrilineal inheritance, and a possible mitochondrial contribution to significant clinical heterogeneity.

## Patients and methods

### Patients

**Fig. 1:**
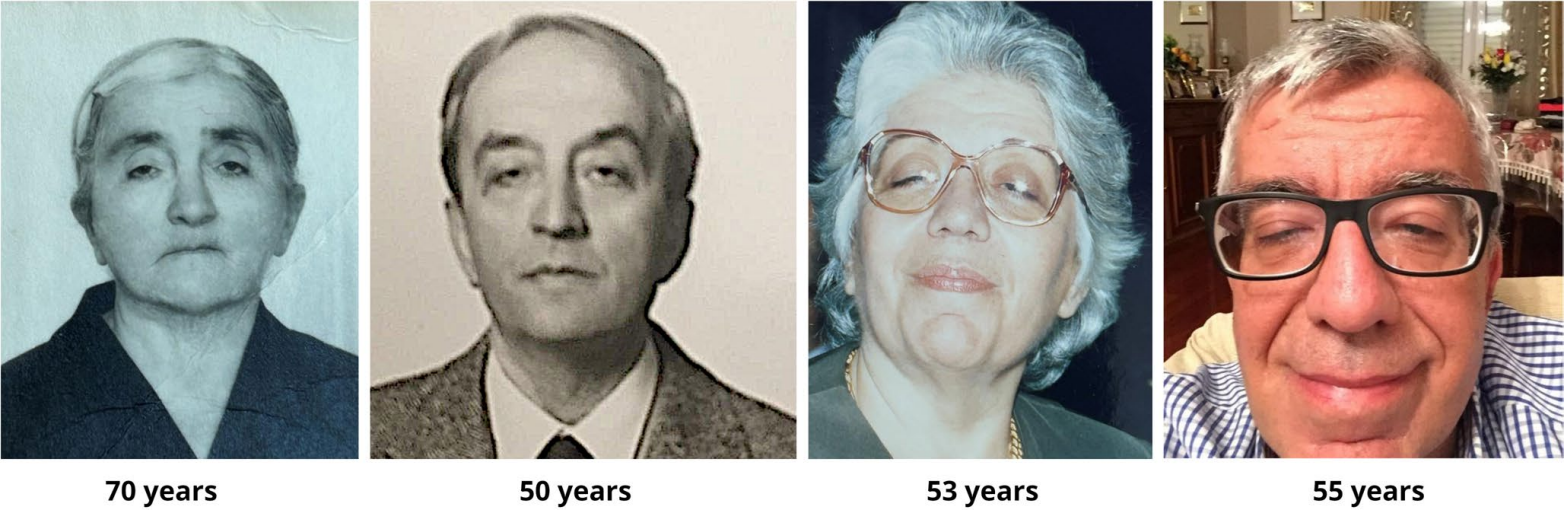
Photographs of Family (F15) members. Three-generation intrafamilial heterogeneity is recorded. The patient’s grandmother (first on the left, age 70 in photo) presented with ptosis, while her son (second from the left, age 50 in photo) developed ptosis at an earlier age. Her daughter—the index patient’s mother (second from the right, age 53 in the photo)—exhibited ptosis, dysphagia, and mild weakness. The index patient (far right, age 55 in the photo) was already experiencing dysphagia, ptosis, and severe walking difficulties

During “2010–2024”, thirty-nine patients (25 males and 14 females) were referred to the Laboratory of Medical Genetics, NKUA, for genetic investigation of ptosis and/or dysphagia in adulthood. Informed consent for genetic and biopsy analysis were obtained. For the purposes of the study, all referring physicians re-evaluated the clinical data, with a particular focus on family pedigrees and providing updated details (Fig. 1). Numerical codes were used to de-identify patients’ details and facilitate retrospective data analysis. Under the Greek legislation and clause 50 of the new EU General Data Protection Regulation (Directive 95/46/EC), no additional consent is required when retrospective studies are performed. The study is part of a project approved by the Scientific and Ethics Committee of “Aghia Sophia” Children’s Hospital (No. 26935/19-12-2019) and was conducted in accordance with the ethical standards as laid down in the 1964 Declaration of Helsinki and its later amendments.

Clinical and demographic data of our probands were collected for each participant. Family pedigrees were based on participants’ descriptions and family photos, particularly for parents and grandparents where genetic confirmation was lacking (Fig. 1).

### PABPN1 genotyping

**Fig. 2:**
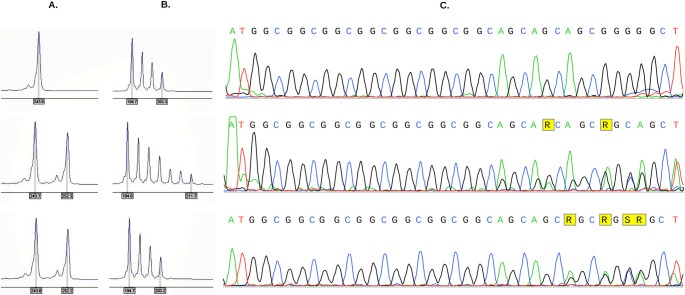
Electropherograms of fluorescence-labeled PCR products showing the presence of expanded alleles in intron 1 of the PABPN1 gene. (**A**) Conventional PCR results. Normal alleles correspond to an amplicon of 244 bp and contain six pure GCG triplets while expanded alleles correspond to an amplicon of 253 bp containing nine pure triplets (**B**) Following TP-PCR, expansions with pure GCG triplets (F15) show a trinucleotide stuttering beyond the normal range while, when GCN are interspersed (F9) no picks out of the normal range are detected. Sanger sequencing confirms the presence of six and nine GCG triplets (**C**)

Genotyping of *PABPN1* (GCN) repeat expansions focused on the evaluation of both the type and number of alanine coding triplets A three-step procedure was followed, which included: i) a fluorescent polymerase chain reaction with primers previously described , ii) a repeat-primed PCR using the specific to codon (GCG) 5΄-ACGCCATCCCAGTTTGAGACGCCGCCGCCGCCGC-3΄ sequence, the 5’- ACGCCATCCCAGTTTGAGACG-3 ‘ universal sequence (stutter amplification patterns were considered indicative of expansions) universal sequence (stutter amplification patterns were considered indicative of expansions) and the labeled 1 F (FAM-CCAGTGCCCCGCCTTAGA-3΄ forward primer sequence and iii) Sanger sequencing of expanded alleles (Fig. 2).

### WES study

WES analysis was performed for patient F15 to investigate potential confounding factors contributing to more severe disease progression. Library preparation was carried out using IDT xGen Exome Research v2 kit (Integrated DNA Technologies). The resulting libraries were subjected to paired-end sequencing on an Illumina NextSeq 500 platform. Variant analysis was performed using VarSome Clinical platform (Saphetor SA) (https://varsome.com/) included CNV detection by ExomeDepth [19] and was based on the phenotype-driven strategy. Variant classification was based on the American College of Medical Genetics and Genomics (ACMG) guidelines [20] and ClinGen recommendations.

### MtDNA study

MtDNA analysis was performed on total DNA extracted from muscle biopsy specimens using the QIAamp^®^ DNA Blood Mini Kit (Cat No 51104). Long-range PCR used F (616–638) and R (15997 − 15975) primers to amplify a wild-type product of 15,382 bp (primer numbers refer to NCBI Reference Sequence NC_012920.1) detected after electrophoresis on a 0.65%/TAE agarose gel. The most common heteroplasmic m.A3243G, variant- associated with mitochondrial diabetes- was specifically studied in patients F9, F17, where early onset diabetes (< 45y) was recorded as a comorbidity (Table 1).

**Table 1:** Summarized clinical, laboratory and genetic findings of the Greek OPMD cohort

Index case	Sex	Age at referral	Age of onset	First symptom	Parental origin/onset	Ptosis	Dysphagia	Muscle weakness	Muscle biopsy	EMG	CPK (IU/L)	COMMENTS	PABPN1 Genotype
**F1**	M^1^	63	58	ptosis	paternal/60	+	-	mild proximal	rimmed vacuoles	denervation of psoas, mild weakness of lower limbs	161	death of his father at 65y^2^ from oesophagus cancer	**(GCN)**_**10**_ **(GCN)**_**13**_ **or** **(GCG)**_**9**_ **(GCA)**_**3**_ **(GCG)**_**1**_
**F2**	F^3^	77	69	ptosis	paternal/60	+	mild	mild	NA^4^	myopathic in biceps and facial muscles	294	coronary disease, death of his father at 86y from a stroke	**(GCN)**_**11**_ **/(GCN)**_**12**_ **(GCG)**_**7**_ **(GCA)**_**3**_ **(GCG)**_**1**_ **/(GCG)**_**7**_ **(GCA)**_**4**_ **(GCG)1**
**F3**	F	56	51	ptosis	paternal/55	+	+(50y)	+	NA	NA	NA	repeated hospitalization and death at 62y due to complications associated with Behcet disease	**(GCN)**_**10**_ **/(GCN)**_**13**_ **(GCG)**_**9**_ **(GCA)**_**3**_ **(GCG)**_**1**_
**F4**	M	77	55	ptosis	paternal/NA	+	+	proximal	rimmed vacuoles	myopathic	mild	ischemic heart disease	**(GCN)**_**10**_ **/(GCN)**_**13**_ **(GCG)**_**9**_ **(GCA)**_**3**_ **(GCG)**_**1**_
**F5**	F	77	60	ptosis	paternal/65	+	+	proximal, scapular winging	NA	myopathic	700	hypothyroidism	**(GCN)**_**10**_ **/(GCN)**_**13**_ **(GCG)**_**6(**_**GCA)**_**1**_**(GCG)**_**2**_**(GCA)**_**3**_**(GCC)**_**1**_
**F6**	F	66	55	ptosis	paternal/60	+	mild	no	NA	NA	normal	nasal voice	**(GCN)**_**10**_ **/(GCN)**_**13**_ **(GCG)**_**6**_**(GCA)**_**1**_**(GCG)**_**2**_**(GCA)**_**3**_**(GCG)**_**1**_
**F7-a**	M	75	52	ptosis	paternal/NA	+	+	mild	NA	NA	NA	death at 77y due to complications associated with influenza	**(GCN)**_**10**_ **/(GCN)**_**14**_ **(GCG)**_**6**_ **(GCA)**_**1**_ **(GCG)**_**3**_ **(GCA)**_**3**_ **(GCG)**_**1**_
**F7-b**	M	52	51	ptosis	paternal/52	+	*+*	-	NA	NA	NA	hypothyroidism deterioration after covid-19 vaccination/disease	**(GCN)**_**10**_ **/GCN)**_**14**_ **(GCG)**_**6**_ **(GCA)**_**1**_ **(GCG)**_**3**_ **(GCA)**_**3**_ **(GCG)**_**1**_
**F8-a**	M	70	63	ptosis	paternal/60	+	+	proximal	NA	myopathic	407	death at 71y from heart attack, death of his father at 65y	**(GCN)**_**10**_ **/(GCN)**_**13**_ **(GCG)**_**9**_ **(GCA)**_**3**_ **(GCG)**_**1**_
**F8-b**	M	73	60	ptosis	paternal/60	+	+	proximal	NA	myopathic	378	myocardial infarction 90y ambulant, brother of F8-a	**(GCN)**_**10**_ **/(GCN)**_**13**_ **(GCG)**_**9**_ **(GCA)**_**3**_ **(GCG)**_**1**_
**F9**	M	62	51	ptosis	paternal/60	+	+	mild weakness (62y)	rimmed vacuoles	NA	NA	diabetes mellitus after 45y like his mother, father and sister with ptosis and dysphagia at 60s	**(GCN)**_**10**_ **/(GCG)**_**13**_ **(GCG)**_**6**_ **(GCA)**_**2**_ **(GCG)**_**1**_ **(GCA)**_**3**_ **(GCG)**_**1**_
**F10**	**M**	54	45	ptosis	paternal/48	+	-	-	-	NA	NA	father with proximal muscle weakness (65y)	**(GCN)**_**10**_ **/(GCN)**_**13**_ **(GCG)**_**9**_ **(GCA)**_**3**_ **(GCG)**_**1**_
**F11**	M	67	~ 52	proximal lower limb, weakness, nasal speech	paternal/54	+	+	Moderate (67y)	rimmed vacuoles, numerous moth-eaten fibers	myopathic, pseudomyotonic discharges	900	severe dysarthria, father, brother and 2 aunts with the same phenotype (onset, symptoms) and death ~ 70s	**(GCN)**_**10**_ **/(GCN)**_**15**_ **(GCG)**_**11**_ **(GCA)**_**3**_ **(GCG)**_**1**_
**F12**	F	69	66	ptosis	paternal/60	+	mild proximal	NA	Normal findings	35	F	chronic urticaria, hypertension and polycythemia vera (JAK2 positive), death of his father at 83y affected by ptosis dysphagia and generalized weakness	**(GCN)**_**10**_ **/(GCN)**_**13**_ **(GCG)**_**9**_ **(GCA)**_**3**_ **(GCG)**_**1**_
**F13-a**	M	72	50	dysphagia	maternal/NA	+	+	proximal (73y)	NA	NA	83	coronary disease, leukoencephalopathy, severe muscle weakness after 3rd dose covid-19 vaccination	**(GCN)**_**10**_ **/(GCN)**_**13**_ **(GCG)**_**9**_ **(GCA)**_**3**_ **(GCG)**_**1**_
**F13-b**	F	76	58	ptosis	maternal/NA	+	-	proximal (72y)	NA	NA	normal	sister of F12-a	**(GCN)**_**10**_ **/(GCN)**_**13**_ **(GCG)**_**9**_ **(GCA)**_**3**_ **(GCG)**_**1**_
**F14-a**	F	62	56	ptosis	maternal/70	+	-	-	NA	NA	NA	gastroesophageal reflux, her mother’s first symptom: dysphagia	**(GCN)**_**10**_ **/(GCN)**_**13**_ **(GCG)**_**9**_ **(GCA)**_**3**_ **(GCG)**_**1**_
**F14-b**	M	43	42	dysphagia	maternal/56	-	+	-	NA	NA	NA	son of F14-a	**(GCN)**_**10**_ **/(GCN)**_**13**_ **(GCG)**_**9**_ **(GCA)**_**3**_ **(GCG)**_**1**_
**F15**	M	52	48	dysphagia	maternal/50	+	+	proximal (52y)	rimmed vacuoles, 2–3% COX(-)	NA	1000	grandmother with ptosis (at 63y) and dysphagia ~ at 70y	**(GCN)**_**10**_ **/(GCN)**_**13**_ **(GCG)**_**9**_ **(GCA)**_**3**_ **(GCG)**_**1**_
**F16**	M	62	50	ptosis	maternal/65	+	+	mild proximal	NA	NA	326	mother's death at 86y	**(GCN)**_**10**_ **/(GCN)**_**13**_ (**GCG)**_**9**_**(GCA)**_**3**_**(GCG)**_**1**_
**F17**	M	69	55	ptosis	maternal/70	+(55)	+(68)	yes (knees) at 60y	NA	NA	912	nasal voice, diabetes mother's death at 104 y	**(GCN)**_**10**_ **/GCN)**_**13**_ **(GCG)**_**9**_ **(GCA)**_**3**_ **(GCG)**_**1**_
**F18**	M	72	70	ptosis	not -affected (deceased ~ 90 y )	+	+	distal	NA	neuromuscular junction dysfunction by Single-fiber EMG	NA	atrial fibrillation	**(GCN)**_**10**_ **/(GCN)**_**12**_ **(GCG)**_**8**_ **(GCA)**_**3**_ **(GCG)**_**1**_
**F19**	M	79	77	ptosis	recessive	+	+	NA	NA	myopathic	normal	hearing loss (~ 70y), diabetes, heart failure, hoarseness	**(GCN)**_**11**_ **/(GCN)**_**11**_

^*1*^*M: male*, ^*2*^*y: years* ^*3*^*F: female*, ^*4*^*NA: not available*

### Muscle biopsy

Muscle biopsy was obtained in three patients and a portion of the muscle sample was snap frozen in liquid-nitrogen-cooled isopentane. Cryostat sections were cut at 6-µm thickness and used for histological, histochemical, and immunohistochemical studies, using conventional techniques. Muscle morphology was assessed using hematoxylin–eosin (HE) and Gomori’s modified trichrome stains. Histochemical reactions included nicotinamide-adenine dinucleotide tetrazolium reductase (NADH-TR), succinate dehydrogenase (SDH), cytochrome oxidase (COX), SDH-COX, ATPase (pH 9.4, 4.3, 4.6), phosphorylase, myoadenylate deaminase (AMPDA), nonspecific esterase (NSE), periodic acid Schiff (PAS), diastase-PAS, and Oil Red O.

### Muscle electron microscopy

Frozen muscle specimens for electron microscopy examinations were fixed with 2% paraformaldehyde – 2,5% glutaraldehyde and post-fixed with 1% osmium tetroxide, both in 0,1 M phosphate buffer (pH 7,4) [21, 22]. Epon thin sections (80 nm thickness) were stained with uranyl acetate and lead citrate and examined by the Jeol JEM-2100 Plus Transmission Electron Microscopy equipped with the Gatan OneView camera.

### Statistical analysis

Quantitative variables (age at onset, age at diagnosis) were expressed as mean ± standard deviation (SD), while the qualitative variable (gender) was reported as frequencies. A paired t-test was conducted to examine the differences in age at onset between probands and their parents. A mixed-effects model was employed to assess the effect of parental origin (maternal vs. paternal) on age at onset. Age at onset for both probands and their respective parents was treated as a repeated measure, with individuals categorized into two levels: P1 (parent) and P2 (proband). Only subjects with complete data (i.e., no missing values) were included in the model. Statistical analysis was restricted to subjects with the common (GCN)₁₃ expansion. This group included 15 parent-offspring pairs (9 paternal and 6 maternal) for whom age at onset data were available. Of the 30 individuals, 15 offspring and one parent had genetically confirmed expansions. For the remaining 14 parents, who exhibited clinical symptoms, the expansion was presumed to be (GCN)₁₃, as transgenerational expansions are not known to occur in OPMD. A two-sided p-value < 0.05 was considered statistically significant. Statistical analyses were performed using StataCorp. 2023. Stata Statistical Software: Release 18. College Station, TX: StataCorp LLC.

## Results

Clinical and demographic data of probands are presented in Table 1. An abnormal *PABPN1* (GCN) expansion was detected in 23 individuals including 16 (69.5%) males and 7 (30.5%) females from 19 families (Table 1). Probands had a mean age at onset of 56 (**±** 7.3) years while the mean age at diagnosis was 66 (**±** 9.6) years.

Significant intrafamilial heterogeneity was recorded in family F15 (Fig. 1); the proband exhibited a severe phenotype with rapidly progressive muscle weakness becoming apparent at the age of 50. By the age of 55 years, he started using walking aids and finally became wheel-chair bound by the age of 64 years, within a decade of symptom onset (Table 1). He also presented dysphagia and proximal lower limb weakness, primarily affecting the pelvic girdle, prior to the age of 48. Notably, he is the only individual in the cohort to require a wheelchair and to exhibit a fully developed phenotype at this age. According to the patient, his mother’s clinical presentation was similar but manifested nearly ten years later than in his case. WES instigated by the severe phenotype in the proband revealed no additional pathogenic/likely pathogenic variants, CNVs included.

**Fig. 3:**
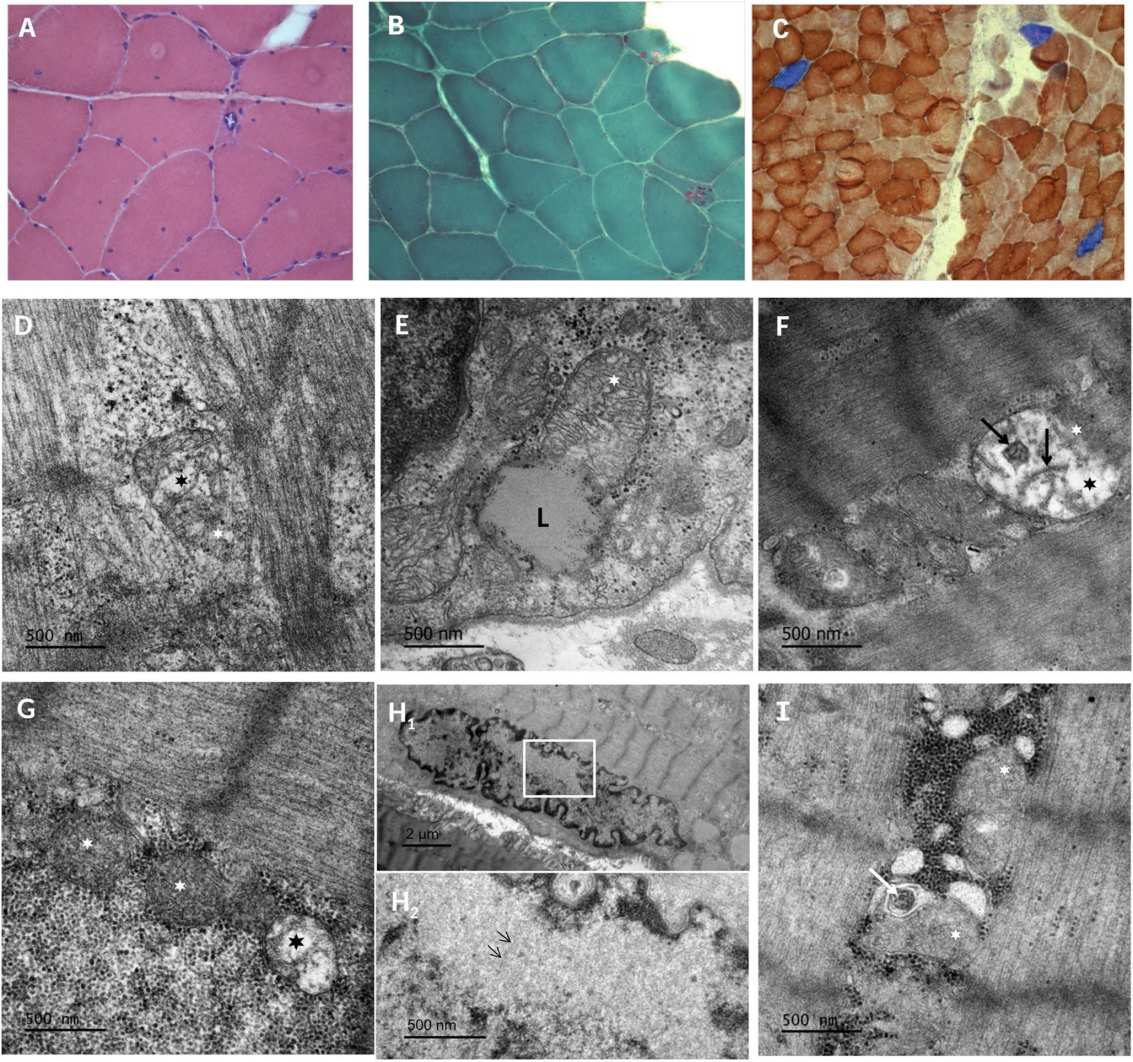
Patient F15: On hematoxylin–eosin (**A**) and modified trichrome Gomori staining (**B)**. Rimmed vacuoles at subcellular or central position usually in atrophic muscle fibers are observed with no ragged red fibers. The COX-SDH double-labeling showed approximately 2–3% COX-negative muscle fibers (**C)**. Similar findings were not recorded in patients F1, F9 (not shown) (**D** – **I)**: Representative electron micrographs on muscle specimens from OPMD patients F15 (**D**, **E**, **F**) F1 (**G**, **H1**, **H2**) and F9 (**I**). Patient F15 exhibits disrupted and enlarged mitochondria with abnormal or poorly defined cristae (**D**, **F**; black asterisks), along with remnants of cristae structures (**F**; black arrows). Electron-lucent material occupying a large portion of mitochondrial area is also observed, indicating atypical accumulation of lipid substances (**L**) inside the matrix (**E**). Patient F1 also presents abnormal mitochondrion with loss of cristae (black asterisk), in close proximity with two normal mitochondria (white asterisks). Patient F9 shows also mitochondria with disrupted arrangement of cristae and the presence of a membranous inclusion in one of them, probably due to cristae aggregation (**I**; white arrow). Irregularly shaped nuclei with multiple invaginations (**H1**) and typical tubulofilamentous material (**H2** in magnification; black thin arrows), known as a typical hallmark of the disease, were observed. Scale bar: 500 nm (**D**, **E**, **F**, **G**, **H2**, **I**) and 2 μm (**H1**)

From the histochemical analysis of 3 muscle biopsies (patients F1, F9, F15), rimmed vacuoles at subcellular or central position usually in atrophic muscle fibers were observed, with no ragged red fibers (Fig. 3A-C). Furthermore, mitochondrial abnormalities were observed, characterized by swelling (Fig. 2D, E, F), disturbed arrangement or partial loss of cristae (Fig. 2D, F, G, I) and atypical lipid inclusions.

**Fig. 4:**
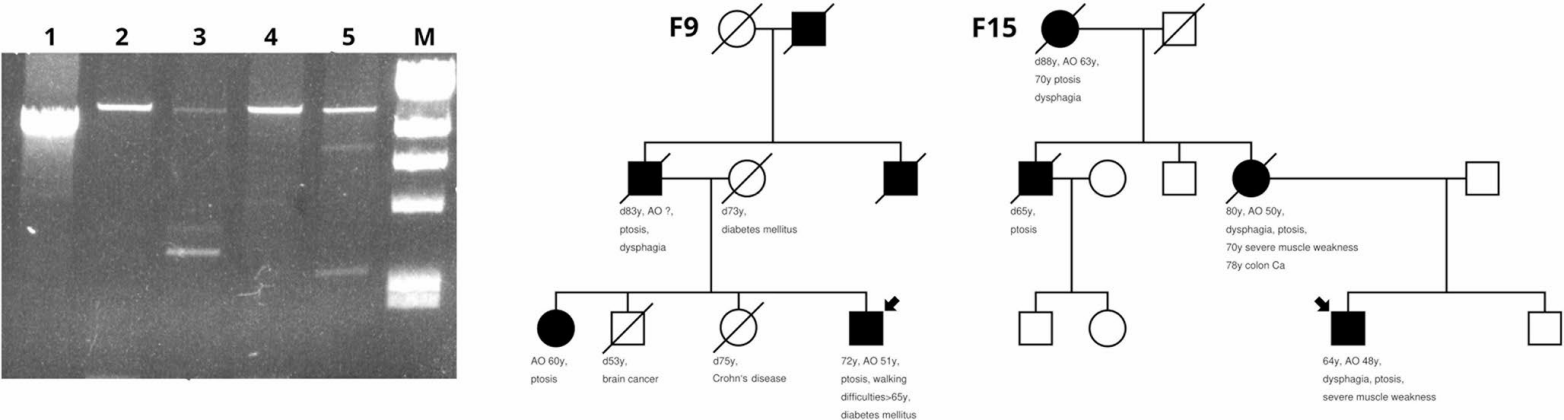
**A**. Agarose gel (0.65% in 1x TAE) electrophoresis of long-range PCR products. The wild-type product is 15,382 bp long. Shorter products indicate the presence of an mtDNA deletion. (1) commonly known deletion (4,977 bp), (2) wild type (3) patient F9 (4) patient F1, (5) patient F15 — M—DNA ladder (λ DNA/HindIII). Samples F9 and F15 are male patients of the same age (52 years) at the time of muscle biopsy and patient F1 is male and was 63 years old at the time of biopsy **B.** Pedigrees of cases F9 and F15 following paternal and maternal inheritance respectively. Although both cases manifest disease symptoms a clear anticipation mode (early onset, disease progression) is recorded. Filled symbols denote clinically affected probands and arrows the index probands. AO: age of onset, y: years, filled boxes: affected members

Analysis of mtDNA extracted from muscle specimens of the same patients showed a wide range of deletions (not common) in the F15 index case (maternal inheritance), but not in age-matched samples including F9 patient (paternal inheritance) (Fig. 4). The m.A3243G variant was excluded in patients F9 and F15, who presented with diabetes.

In total, 8 distinct genotypes were observed in this cohort (Table 2). The common (GCN)_13_ genotype was recorded in 11 families (Table 2). Milder phenotypes were recorded in two patients (F2, F18, F19) with smaller expansions (GCG)_10_/GCG)_12,_ (GCG)_11_/GCG)_12_ and (GCG)_11_/GCG)_11_ accordingly. The most severe presentation was in F15 where the (GCG)_10_/GCG)_13_ genotype was maternally inherited across three generations presenting clinical deterioration. The NM_00463.4: c.30_32dup, p.(Ala11dup) (rs1401656265) known as modifier variant (GCG)_11_, was evaluated in the context of population frequency through mining the Laboratory of Medical Genetics in-house Exome Sequencing (ES) Database of the (LMG-db). The database comprises 1000 unrelated individuals of Greek origin and the variant was detected in 3/1000 individuals, indicating a MAF (Minor Allele Frequency) of 0,003 (0,3%) (Table 1).

**Table 2:** Different genotypes were identified among 19 not related families

No	Genotype	Number of families
1	(GCN)_10_/(GCG)_9_(GCA)_3_(GCG)_1_	11/19
2	(GCN)_10_/(GCG)_6_(GCA)_1_(GCG)_2_(GCA)_3_(GCC)_1_	2/19
3	(GCN)_11_/(GCG)_7_(GCA)_4_(GCG)_1_	1/19
4	(GCN)_10_/(GCG)_6_(GCA)_1_(GCG)_3_(GCA)_3_(GCG)_1_	1/19
5	(GCN)_10_/(GCG)_11_(GCA)_3_(GCG)_1_	1/19
6	(GCN)_10_/(GCG)_8_(GCA)_3_(GCG)_1_	1/19
7	(GCN)_10_/(GCG)_6_(GCA)_2_(GCG)_1_(GCA)_3_(GCG)_1_	1/19
8	(GCN)_11_/(GCG)_11_	1/19

The mean age of onset was 59.1 years (95% CI: 55.9–62.3 years) in affected parents, and 54.8 years (95% CI: 45.1–58.5 years) in offspring. A comparison of age of onset between probands and their parents showed a mean decrease of 4.3% in the offspring group compared to their parents (*p* = 0.025). A statistically significant interaction was identified between individual status (P1: parent, P2: proband) and parental origin (*p* < 0.001). Specifically, individuals with maternally inherited expansions exhibited a greater reduction in age of onset compared to those with paternally inherited (12.0 years vs. 1.1 years).

## Discussion

This study presents OPMD patients of Greek descent with genetic confirmation from a specialized diagnostic center. The geographical origins and/or diverse genotypes observed in the cohort do not support a potential founder effect. Underdiagnosis or delayed diagnosis was observed, due to the rarity and heterogeneity of the disease hindering prompt referral.

Both inter- and intrafamilial clinical heterogeneity were observed, consistent with previous studies [23–25]. Interfamilial heterogeneity was noted in respect to the size of PABPN1 expansions whereby smaller alleles lead to less severe phenotypes [26]. A genetic modifier nature was proposed for the (GCN)_11_ allele when Brais et al. compared compound heterozygotes of the (GCN)_11_/(GCN)_13_ genotype to those with the (GCN)_10_/(GCN)_13_ genotype [1]. In consistence, mild phenotypes were recorded in three of the current cohort patients with (GCN)11/(GCN)11, (GCN)11/(GCN)12 and (GCN)10/(GCN)12 genotypes respectively. In the largest cohort of 354 OPMD patients Richard et al. (2017) (10), observed that patients with expansions greater than (GCN)14, frequently presented with proximal weakness of the pelvic girdle at the time of diagnosis, while other studies support that large expansions are not necessarily linked to severe disease progression [26, 27]. In line with previous Caucasian cohorts, ptosis was the most common initial symptom, but patients presenting with dysphagia as the first symptom were also recorded and linked to maternal inheritance and a much earlier onset compared to those with ptosis [24, 25].

Multiple lines of evidence in model organisms and humans showed that sarcopenia (progressive loss of muscle mass and strength) is one important sign of human aging and that impaired mitochondrial function can contribute to age-associated disease phenotypes and aging [28]. In this context RNA expression studies indicated high similarities between muscles from OPMD patients and elderly subjects indicating that OPMD may be related to an accelerated muscle aging mechanism especially if compared to other myopathies [13]. Abnormal localization of PABPN1 on the inner membrane of mitochondria in the muscle fibers of transgenic mice and cell lines is proposed to involve mitochondrial dysfunction, supported by both the observed mitochondrial dysfunction in OPMD and the critical role of mitochondrial metabolism in muscle cells [17]. This was accompanied by reduced expression of oxidative phosphorylation (OXPHOS) complexes in transgenic mouse and cell models of OPMD, and mitochondrial abnormalities were closely associated with muscle degeneration. Notably, these mitochondrial alterations occurred despite preserved mitochondrial content, indicating a qualitative rather than quantitative defect. In addition, loss-of-function studies by Vest et al. (2017) suggested that reduced levels of wild-type PABPN1 may independently contribute to disease progression [18]. Muscle biopsy availability is scarce, limiting the number of biopsies studied in this cohort to just three. However, ultrastructural findings from skeletal muscle biopsies did reveal mitochondrial abnormalities. The most profound included enlarged mitochondria with sparse cristae and lipid accumulation, and concerned the sample from patient F15, a member of a family with a three generation matrilinear inheritance, characterized with the most severe clinical presentation. These findings are consistent with age-related mitochondrial alterations and associated skeletal muscle decline [29]. Mitochondrial abnormalities were first described in 1995 by Wong et al., who noticed fibers containing paracrystalline mitochondrial inclusions on muscle biopsy electron microscopy form a male patient (onset at 41 years) and his mother (onset at 55) [30]. Mugit et al. commented on monozygotic twins with identical PABPN1 expansions and mtDNA deletions, who also exhibited accumulations of abnormal mitochondria and characteristic ‘‘parking lot’’ inclusions observed on electron micrographs [31]. The patients showed bilateral ptosis, progressive weakness, large joint problems, dysphagia and deafness. The expanded allele was transmitted by their mother and grandmother who were reported only with bilateral ptosis [31].

The debate regarding anticipation, meaning earlier age or greater severity of symptoms, in consecutive generations of OPMD pedigrees is still ongoing [23, 32]. Blumen et al. were the first to report an anticipation effect in 1993, when 26 out of 29 pediatric patients were recorded to exhibit aggravated clinical presentations compared to their parents examined at the same time. However, no genetic findings or details about patients/pedigrees were available [33]. In other trinucleotide-repeat expansion disorders, such as myotonic dystrophy and Huntington’s disease, deterioration of the phenotype and earlier disease onset is attributed to the number of repeats exceeding a critical threshold through a dynamic transmission from parents to offspring. In the case of OPMD, many studies on segregation and inheritance indicate that the number of (GCN) triplets remains stable through multiple generations of parents-offspring pairs. In the current cohort anticipation was recorded in 6 cases (including the severe F15 case). All involve matrilineal transmission and at least 10 years decrease in the age of onset followed by an aggravation of disease complications. These findings, although very limited, are in line with those of previous studies of intrafamilial heterogeneity, favoring true anticipation in OPMD especially in families with matrilineal transmission in over two generations. A possible explanation could be the effect of deregulated mitochondria inherited through the maternal lineage, due to the disease’s impact on mitochondrial integrity. Affected maternal mitochondria mediate to the deterioration of disease, perceived as an idiosyncratic form of anticipation effect proposed to be named “mitodrasis” from the combination of the Greek words “mito-chondria” and “drasis” which means “action”. Elías-López et al. (2023) reviewed evidence suggesting that damaged mitochondria may be transmitted to the second and third generations in mice similar to findings from studies in which obese mothers transmitted abnormal mitochondria through their oocytes [34]. The impact of maternal inheritance may be further illustrated by findings from additional genetic disorders. In myotonic dystrophy type 2 although no correlation between disease severity and CCTG repeat length in intron 1 of the *CNBP* gene is indicated, it seems that maternal inheritance may increase the risk for disease early onset, subsequent complications, and cardiovascular events [35]. In further support of the matrilineal impact, Tileman et al., (2022) described an unusual case of a pediatric DM2 patient with an abnormal expansion similar to that of her mother, maternal uncle, and grandmother (CCTG expansions 25 to > 2000 repeats in each patient) [36]. Recent proteomic and transcriptomic analyses revealed a downregulation of essential mitochondrial proteins in muscle biopsies from patients with DM2 also revealing widespread ultrastructural mitochondrial abnormalities, including dysmorphic mitochondria with paracrystalline inclusions on electron microscopy [37]. In Barakat syndrome, a rare genetic disorder also known as Hypoparathyroidism, Sensorineural Deafness and Renal disease (HDR syndrome), a genetic anticipation effect was envisioned by Wang et al. (2017) as a possible explanation for transgenerational phenotypic heterogeneity [38]. The related *GATA3* gene encodes for a transcription factor involved in embryonic development and mitochondrial biogenesis [39]. In Alexander Disease (AxD), a rare autosomal dominant leukodystrophy caused by missense variants in glial fibrillary acidic protein (*GFAP*), a maternal-origin effect was suspected to result in an earlier age of onset in the offspring in 25 out of 33 cases reviewed by Hunt et al. (2021) [40]. GFAP is the major intermediate filament of astrocytes in the central nervous system, the loss of which results in hyperfused mitochondria.

Although a mitochondrial contribution cannot be excluded, the primary limitation of the ‘mitodrasis-’ hypothesis lies in the stochastic nature of the findings, driven by the small cohort size, which consequently limits statistical power. Given the scarce literature data on disease onset and progression in previous generations, our findings—while preliminary—may provide a novel perspective to guide future research. The retrospective design of this study, along with potential ascertainment bias during anamnesis, should also be considered, as increased awareness of the disease in more recent generations may have influenced reporting and diagnosis. Since abnormal mitochondria can result either from defective mitochondrial DNA (mtDNA) or pathogenic nuclear variants, the combination of these defects may explain the reduced mitochondrial activity observed in maternal transgenerational transmissions. In this context, although the maternal-origin of affected nuclear alleles in combination with additional mtDNA pathogenic variants, might explain increased intrafamilial variability due to tissue specificity and heteroplasmy, it cannot explain anticipation. Factors dependent on female sex, e.g. hormone profiles, may ameliorate symptoms in female patients but do not justify the earlier onset or severe progression observed in female offspring. Whether the observed anticipation in OPMD, as well as in other not related to dynamic expansions diseases, is a true phenomenon requires addressing the presence of multiple anticipation factors. Zheng et al. used high-throughput methylation profile analyses to discover that DNA methylation changes at the 4q35 D4Z4 repeat locus correlate with clinical severity and generational onset of FSHD1, supporting epigenetic effects on genetic anticipation in a muscular dystrophy context [41]. Studies using Drosophilla models also showed mitochondrial dysfunction due to mRNA Poly(A) Tail Regulation in OPMD but more studies, with patient transcriptome analysis, are needed to clarify a similar impact in humans [9].

## Conclusions

Although genotype analysis in OPMD can distinguish between mild and severe cases based on the number of expanded alanine repeats, it does not fully explain the observed intrafamilial phenotypic heterogeneity. Comprehensive approaches, such as long-read sequencing -where structural variants and repeats may be assessed -and epigenetic profiling, may help identify additional factors contributing to the genotype–phenotype correlation. In the case of deterioration through maternal transmission, further experimental confirmations combined with observations from the pedigrees, histological and ultrastructural data, and mtDNA analysis may help to evaluate if that intrafamilial variability could be the cumulative effect of mitochondrial dysfunction through generations. Assessing the presence of anticipation in other OPMD cohorts or other genetic diseases would be useful in confirming whether “mitodrasis” is a real phenomenon. This could provide therapeutic options through the restoration or augmentation of mitochondrial function, as no treatments are currently available for OPMD disease to date.

## Data Availability

No datasets were generated or analysed during the current study.
